# Factors accounting for the association between anxiety and depression, and eczema: the Hordaland health study (HUSK)

**DOI:** 10.1186/1471-5945-10-3

**Published:** 2010-04-22

**Authors:** Marianne Klokk, Karl Gunnar Gotestam, Arnstein Mykletun

**Affiliations:** 1 Department of Adult Psychiatry, Aalesund Hospital, Helse Sunnmore HF, N-6026 Aalesund, Norway; 2 Department of Neuroscience, Norwegian University of Science and Technology, MTFS, NO-7489 Trondheim, Norway; 3 Research Centre for Health Promotion (HEMIL), Faculty of Psychology, University of Bergen, Christiesgt. 13, N-5015 Bergen, Norway; 4 Division of Mental Health, Norwegian Institute of Public Health, Pb 4404 Nydalen, NO-0403 Oslo, Norway

## Abstract

**Background:**

The association between anxiety and depression, and eczema is well known in the literature, but factors underlying this association remain unclear. Low levels of omega-3 fatty acids and female gender have been found to be associated with both depression and eczema. Somatization and health anxiety are known to be associated with anxiety and depression, further, somatization symptoms and health anxiety have also been found in several dermatological conditions. Accordingly, omega-3 fatty acid supplement, female gender, somatization and health anxiety are possible contributing factors in the association between anxiety and depression, and eczema. The aim of the study is to examine the relevance of proposed contributing factors for the association between anxiety and depression, and eczema, including, omega-3 fatty acid supplement, female gender, health anxiety and somatization.

**Methods:**

Anxiety and depression was measured in the general population (n = 15715) employing the Hospital Anxiety and Depression Scale (HADS). Information on eczema, female gender, omega-3 fatty acid supplement, health anxiety and somatization was obtained by self-report.

**Results:**

Somatization and health anxiety accounted for more than half of the association between anxiety/depression, and eczema, while the other factors examined were of minor relevance for the association of interest.

**Conclusions:**

We found no support for female gender and omega-3 fatty acid supplement as contributing factors in the association between anxiety/depression, and eczema. Somatization and health anxiety accounted for about half of the association between anxiety/depression, and eczema, somatization contributed most. The association between anxiety/depression, and eczema was insignificant after adjustment for somatization and health anxiety. Biological mechanisms underlying the mediating effect of somatization are yet to be revealed.

## Background

The field of psychodermatology addresses the interaction between skin and mind, and the association between anxiety and depression, and eczema is well documented in the literature [1-8]. However, despite of numerous hypotheses, there is still surprisingly little empirical evidence for mechanisms involved in this association.

Omega-3 fatty acid level has been suggested to be a possible contributing factor in the association between depression and atopic eczema [7]. This hypothesis is inspired by reports of low levels of omega-3 fatty acids in blood and development of atopic disease [9], and reports of low dietary intake of omega-3 fatty acids and depression [10-12].

Timonen and colleagues have suggested that the association between atopic disorders and depression is limited to females only. This is based on their findings in two large, population-based studies, where women suffering from atopic diseases, compared with nonatopic subjects, were found to possess an elevated risk for developing depression [7,13]. A corresponding association was not found among males. Timonen and colleagues also propose that parity and female hormones may contribute in the association between depression and atopic disorders such as eczema [7,13]. This is based on reports of associations between parity and gestational age, and atopic disorders [14], and associations between multiparity, atopy, and depression [15].

A substantial part of the psychodermatological literature has focused on atopic forms of eczema, and many of the above referred hypotheses concerning possible explanatory factors for the association between anxiety and depression, and eczema presuppose the involvement of atopy (elevated levels of IgE, in addition to "atopic symptoms" [16]) as a part of the aetiological chain underlying this association. On this basis, we considered IgE levels as a possible explanatory factor in the association between anxiety, depression, and eczema, but we recently published that there is no association between IgE levels, and anxiety and depression [17]. On the basis of this nil finding, IgE levels cannot be a mediating or confounding factor in the association of interest. Therefore, we decided not to include analyses of IgE in this manuscript.

According to Koo and Lebwohl, disfiguring skin conditions can lead to psychological problems such as decreased self-esteem, that may lead to depression or symptoms of social phobia [1]. Several reports have emphasized psychological mechanisms beyond anxiety and depression, that may contribute in eczema: Eczema may be worsened by emotional stress [1], and some reports claim that eczema patients are more prone to express psychosomatic symptoms than normal controls [18]. According to a recent review by Gupta, a wide range of somatization-related symptoms are found in dermatological patients [19]. A according to the World Health Organisation (WHO)'s International Classification of Diseases, 10th revision (ICD-10), somatization is a mental disorder characterized by experience of somatic symptoms without known organic etiology, where symptoms often spread across organ systems, and vary over time [20]. Whereas, somatization disorder is rare in the general population, sub-clinical tendency of somatization is common. There are also reports of higher prevalence of hypochondria symptoms in eczema patients than in normal controls [21]. Hypochondria is an old clinical concept referring to exaggerated health anxiety (health-related worry and fear) and inadequate beliefs of suffering from a serious medical disease [22]. It is well known that anxiety and depression often are associated with both somatization and health anxiety [23-25], but, according to ICD-10, comorbidity with anxiety and/or depression is not a criterion for the diagnosis of somatization disorder. Due to the reported associations between eczema, and anxiety, depression, somatization and health anxiety, it is possible to suggest that somatization and health anxiety could be contributing factors in the association between anxiety and depression, and eczema, but as far as we know, this has not been previously investigated.

Most studies within the field of psychodermatology are based on clinical samples, commonly recruited from dermatological clinics. Such studies are often criticized for low statistical power and multiple selection biases. On the other hand, the advantage of clinical studies is the presumed good reliability of diagnoses of eczema, anxiety, and depression. Compared to clinical studies, epidemiological population-based studies are less vulnerable to selection bias and are commonly more generously powered. There are few epidemiological population-based studies within the psychodermatological field; among the exceptions are Timonen and colleagues [7,8,13,26].

Despite the broad range of hypotheses concerning the association between anxiety and depression, and eczema, empirical support is scarce for most of them. The aim of the present study is therefore to examine the relevance of proposed contributing factors for the association between anxiety and depression, and eczema, including omega-3 fatty acid supplement, female gender, health anxiety, and somatization.

## Methods

### Participants and data collection

The Hordaland Health Study (HUSK) was conducted as collaboration between the National Health Screening Service, the University of Bergen and local health services of Hordaland County in Norway. All individuals born 1953-57, who resided in Hordaland County on December 31, 1997, were invited to the HUSK study. The target population counted 29400 individuals [27]. A total of 18581 (8598 men and 9983 women) both answered the first questionnaire and came to clinical examination. A random sample of about 6300 individuals born 1950-1951, that participated in a previous (1992-93) health study in Hordaland, were also invited. Finally, 18581 individuals born 1953-57 (participation rate of 63%), and 4849 individuals born 1950-51 (participation rate of 77%) attended. In the present study, we included only those who came to clinical examination, and returned the second self-report form, further excluding those without valid responses on eczema and HADS variables (about 33% of the participants). Thus the final population consisted of 15 715 individuals.

Data collection in HUSK was performed over two steps: First, participants were mailed a questionnaire and an invitation to a health examination (measuring blood pressure, height, weight and hip-waist ratio), where also blood samples were taken. After handing in the first form at the examination, the participants were given one of two versions of a second form to be returned by post in a prepaid envelope [27].

### Measures

#### Anxiety and depression

The Hospital Anxiety and Depression Scale (HADS) is a self-report questionnaire comprising 14 four-point scaled items, seven for anxiety (HADS-A) and seven for depression (HADS-D) [28]. To avoid false positive cases in context of somatic diseases, no somatic items or items regarding sleeping difficulties are included. In accordance with the findings on anxiety disorders and depression in the National Comorbidity Survey [29], the HADS components of anxiety and depression shares 30% variance [30]. According to a recent literature review covering 31 studies, HADS has shown good case-finding properties for anxiety and depression in patient populations in primary care and hospital settings [31]. A cut-off score of 8 on both subscales was found to give an optimal balance between sensitivity and specificity, both at about 0.80, for depression and anxiety according to DSM-III and IV, ICD-8 and 9. This is similar to the sensitivity and specificity of the General Health Questionnaire. According to previous use of the HADS scale, anxiety and depression were operationalized in two ways: By employing cut-offs (cut-off score of 8 on both subscales), four groups were identified as current anxiety only, current depression only, current comorbid anxiety and depression, and a reference group (no disorder) [32,33]. Further, anxiety and depression symptom load was measured continuously employing HADS anxiety and depression sum-scores [17].

#### Eczema

The eczema diagnosis in HUSK was based on self-report questions extracted from three epidemiological studies concerning hand eczema [34-36]. The use of self-report for eczema has been validated against dermatologists' diagnosis [37,38]. The self report question "have you had hand eczema on any occasion during the past 12 months?" was validated in different occupational groups [38] and resulted in 10-12.5% false negative cases compared to diagnoses established by dermatologist. This corresponds to a sensitivity of 53-59% and specificity of 96-99%.

HUSK contained dichotomous questions about eczema similar to the self-report question used in the above referred validation study: "Have you had eczema (red, itching, sensitive, or fissured skin) on any occasion during the last year?" Responses were recorded with reference to hands, face, and body. The eczema variables were operationalized as any eczema, number of areas affected, and hand, body and face eczema separately. A broader use of the eczema concept has been suggested in the revised nomenclature for allergy for global use. According to this nomenclature, the term eczema is considered to be a general term comprising both atopic and non-atopic eczemas, further sub-classification of eczema will require determination of underlying immunological mechanism [16]. However, in the current study eczema questions are general and further sub-classification beyond report of body area involved is restricted.

#### Socio-demographic variables and health related behaviour

Socio-economic factors and health-related behaviours are shown to be associated with both skin disease [39] and anxiety and depression [40,41], thus socio-economic factors might confound the association of interest. In addition, female gender has been suggested by Timonen and colleagues to moderate the association between eczema and depression [7,13]. Consequently, we adjusted for gender (data from public registries), marital status (living with a partner or not), current smoking (dichotomy), household income (three levels), education (three levels), and main occupation and industry, classified according to Standard Classification of Occupations (ISCO88), and Standard Industrial Classification (SIC94) [27]. Different kinds of "wet work" (for instance work in health care sector, hair dressers or cooks) are also among the occupations included in this classification.

#### Physical health in HUSK

Somatic diagnoses of stroke, myocardial infarction, angina pectoris, multiple sclerosis, asthma, diabetes mellitus, hay-fever, bronchitis, osteoporosis, and fibromyalgia were registered as dichotomies. In addition, all current pharmacological treatments were registered in open questions, also with responses for which somatic diagnoses treatments were used for [42]. Common responses like dietary supplements and alternative pharmacological treatments were not included. From this list 131 common, non-acute conditions were identified, including haematological, cardiovascular, gastrointestinal, urogenital, hormonal, neurological, respiratory, neoplastic, immunologic, and infectious diseases. All responses were encoded according to the Anatomical Therapeutic Chemical Classification System (ATC) [43], and the International Classification of Primary Care (ICPC) [44], by a scientific committee. An index was computed as the number of somatic diagnoses based on the above information (9 self-reported somatic diagnoses in addition to 131 somatic conditions that involve pharmacological treatment). As the diagnosis of fibromyalgia is symptom-based, thus touching on to the construct of somatization, we included it as a separate dichotomy (as opposed to be included in the indexes).

#### Somatization and health anxiety (Whiteley Index), measured in sub-sample I (n = 7024)

In HUSK, participants were asked to indicate any presence of 13 somatic symptoms from the ICD-10 symptom criteria for F45.0 somatization disorder, and rate them as to frequency scaled from 0 (almost never) to 4 (all the time). One of these involved skin symptoms and was omitted in this context to avoid circularity. To conserve variance in the data, a mean score of responses across the remaining 12 items was computed for every individual, and the mean score represents "somatization" in the individual. This somatization symptom score will, however, be sensitive to organic aetiology. To make the index more specific to symptoms relating to somatization, we adjust all analyses for somatic diagnoses and medication for somatic conditions (described above) before including somatization in the models. Our Index for somatization should not be compared to validated instruments for the diagnosis of somatization disorder. Our index for somatization was used as a dimensional measure (using a mean-score of the 12 items) for tendency of somatization. However, this dimensional measure for somatization has also been used in previous publications [33,45].

The Whiteley Index [46] is a self-report scale on hypochondria and health anxiety consisting of 14 items, and has sensitivity and specificity rates of 71% and 80% for the DSM-IV definition of hypochondria [22,45]. The Whiteley Index has been found to be a valid screening instrument in the assessment of hypochondria disorder [22], but in this study it was used dimensionally to allow maximum variance and we describe it under the term "health anxiety"[45]. In HUSK, the Whiteley Index was distributed to a sub-sample only; hence adjustments for health anxiety were performed in a sub-sample including 7024 individuals (sub-sample I).

#### Omega-3 fatty acid supplement

Cod liver oil (containing on average 1.2 gram omega-3 fatty-acids per 5 ml) supplement was measured by self-report of numbers of months with daily cod liver oil intake, during the last year.

#### Hormonal replacement therapy, hormonal contraceptives, and parity

For women, we included self-report of parity (0 - 5 or more children), current use of hormonal replacement therapy (HRT) (Oestrogen tablets or plaster), and current use of hormonal contraceptives (P-pill, or Mini-pill or Hormone-spiral).

### Statistical analysis

Sample characteristics were examined by descriptive statistics. Chi-square statistics were applied when exploring differences in sample characteristics between men and women. Pearson's correlation coefficients were calculated to examine correlations between health anxiety, and somatization, and between anxiety/depression, and health anxiety and somatization.

To test hypotheses of associations between anxiety and depression, and eczema, logistic regression analyses were applied. Anxiety, depression, and co-morbid anxiety/depression were included as dependent variables, and eczema as independent variable. Linear regression was used to compare the strength of associations between eczema and somatization, eczema and health anxiety, and eczema and anxiety/depression. Effects were given as Odds Ratios (OR) for logistic regressions, and unstandardized regression coefficients (B) for linear regressions. All results were reported with 95% confidence intervals. The proposed confounders and possible explanatory factors of the association between anxiety/depression, and eczema were included in the regression model as covariates in both bi- and multivariate logistic regression analyses. No automatic procedures for including/excluding variables in the regression model were used.

Age was not regarded a relevant confounder.

### Ethics

The study was approved by the Regional Committee for Medical Research Ethics of Western Norway and the Norwegian Data Inspectorate.

## Results

Descriptive characteristics of the study sample are reported in Table 1. Any eczema was reported among 36% of the sample, distributed on hand eczema (20.2%), body eczema (19.8%), and face eczema (9.2%). Both hand eczema (24.2% in women vs. 15.4% in men) and face eczema (9.9% vs.8.3%) were more prevalent in women than in men (p < 0.01). The prevalence of body eczema was nearly equal in men and women (about 20%). According to the HADS definitions, 12.9% had current anxiety only, 4.2% current depression only, and 7.5% co-morbid anxiety/depression.

**Table 1:** Descriptive characteristics.

Variables	N at risk	Percent
**Age**		
Mean	43.0	
SD	1.41	
Range	41-48	
**Gender **(males)	7115	45.3
**Married/living with a partner **(yes)	11718	74.6
**Current smoking **(yes)	5424	34.5
Household Income		
0-17800 EUR	857	5.5
17815-47490 EUR	7122	45.3
47500 EUR or more	7086	45.1
**Education**		
Compulsory only	2871	18.3
Secondary, non-university	7193	45.8
University	5651	36.0
Physical health*		
No diagnosis	11203	71.3
1 diagnosis	3253	20.7
2 diagnoses	889	5.7
3 or more	370	2.4
**Fibromyalgia **(yes)	1053	6.7
**Parity^1^**		
0 children	699	8.1
1 or more children [combined in this table only]	7732	89.9
**Hormonal contraceptives^2 ^**(yes)	1672	19.4
**HRT^1 ^**(yes)	414	4.8

SD, standard deviation; HRT, Hormonal replacement therapy (Oestrogen tablets or plaster);

EUR, Euro.

*Self-reported somatic diagnoses and medication for somatic conditions

^1^Women only (N = 8600).

### Associations between anxiety and depression, and eczema

Anxiety, depression, and co-morbid anxiety/depression were associated with eczema across all body areas, the exception being face eczema which was not associated with depression in the absence of anxiety. Co-morbid anxiety/depression had a stronger association to eczema than current anxiety and depression only. The strongest association was found between face eczema and co-morbid anxiety/depression (Table 2, upper part).

**Table 2:** Associations between anxiety and depression, and eczema.

			Anxiety		Depression		Both	
	
			OR	95%CI	OR	95%CI	OR	95%CI
**Men and women**								
Any eczema			1.49*	1.35-1.64	1.25*	1.04-1.49	1.55*	1.36-1.76
Hand eczema			1.41*	1.26-1.59	1.26*	1.02-1.54	1.32*	1.13-1.53
Face eczema			1.54*	1.32-1.79	1.27	0.96-1.69	1.67*	1.37-2.03
Body eczema			1.47*	1.31-1.65	1.43*	1.17-1.75	1.59*	1.37-1.84
**Women**								
Any eczema			1.40*	1.23-1.58	1.16	0.88-1.53	1.66*	1.39-1.98
Hand eczema			1.28*	1.11-1.47	1.07	0.78-1.47	1.25*	1.02-1.52
Face eczema			1.41*	1.16-1.71	0.97	0.60-1.60	1.66*	1.29-2.14
Body eczema			1.49*	1.29-1.73	1.44*	1.05-1.98	1.59*	1.30-1.94
**Men**								
Any eczema			1.54*	1.30-1.81	1.42*	1.13-1.78	1.42*	1.16-1.73
Hand eczema			1.49*	1.22-1.84	1.69*	1.29-2.21	1.44*	1.12-1.84
Face eczema			1.71*	1.33-2.21	1.59*	1.12-2.28	1.67*	1.23-2.26
Body eczema			1.44*	1.19-1.74	1.43*	1.10-1.85	1.59*	1.28-1.98
**Interaction terms**								
Any eczema by gender			0.91	0.74-1.12	0.82	0.57-1.18	1.17	0.90-1.52
Hand eczema by gender			0.86	0.67-1.10	0.63*	0.42-0.96	0.87	0.63-1.19
Face eczema by gender			0.82	0.60-1.13	0.61	0.34-1.11	1.00	0.67-1.48
Body eczema by gender			1.04	0.82-1.32	1.01	0.67-1.52	1.00	0.74-1.35

OR, odds ratio; CI, confidence interval. *p < 0.05.

One eczema by gender interaction was found to be statistically significant in association with anxiety/depression (Table 2, bottom part), this result is probably due to statistical chance across 12 interaction analyses, because with a p-value that was 0.03 for this analysis, only by setting the level of significance at p = 0.025, this interaction will turn out insignificant. Accordingly, this gender interaction would not be robust for a Bonferroni correction.

Analyses were also carried out for "pure" forms of hand, face and body eczema (that is, not co-occurring with eczema in other body areas) and anxiety/depression, giving principally the same results as in the initial analyses, but generally with weaker associations (results not shown in table). A dose-response relationship between number of areas with eczema and anxiety/depression was found (results not shown in table).

### Somatization, health anxiety, fibromyalgia and somatic diagnoses

Adjustment for the somatization and health anxiety together represented about half the association between anxiety and depression, and eczema (Table 3, sub-sample I), somatization being a stronger factor here than health anxiety. Pearson correlation between somatization and health anxiety was considerable (r = 0.49, p < 0.01), thus their explanatory effect for the association is partly overlapping.

**Table 3:** Anxiety and depression as a function of eczema, with cumulative adjustments for possible contributing factors.

	Anxiety		Depression		Both	
	
Cumulative adjustments	OR	95%CI	OR	95%CI	OR	95%CI
**Total sample (N = 15715)**						
None	1.49*	1.35-1.64	1.25*	1.04-1.49	1.55*	1.36-1.76
Gender	1.45*	1.31-1.60	1.31*	1.09-1.56	1.55*	1.36-1.77
SES^1^	1.43*	1.30-1.59	1.28*	1.07-1.53	1.52*	1.33-1.73
Occupation/Industry	1.43*	1.30-1.59	1.26*	1.05-1.51	1.50*	1.31-1.71
Omega-3 supplement	1.42*	1.29-1.58	1.26*	1.06-1.51	1.50*	1.31-1.71
Current smoking	1.43*	1.26-1.51	1.26*	1.05-1.51	1.50*	1.31-1.72
**Subsample I (N = 7024)**						
None	1.45*	1.24-1.69	1.41*	1.10-1.81	1.60*	1.32-1.95
Gender and SES^1^	1.40*	1.19-1.63	1.43*	1.11-1.84	1.56*	1.27-1.90
Fibromyalgia	1.37*	1.17-1.60	1.40*	1.08-1.80	1.51*	1.24-1.85
Other somatic diagnoses^2^	1.34*	1.15-1.57	1.40*	1.08-1.80	1.47*	1.20-1.80
Somatization^3^	1.15	0.98-1.35	1.27	0.98-1.64	1.21	0.97-1.50
Health anxiety	1.10	0.93-1.31	1.23	0.95-1.60	1.17	0.93-1.47
Adjusted for all factors above^4^	1.09	0.92-1.29	1.22	0.94-1.59	1.19	0.94-1.51
**Female sub-sample (N = 8600)**						
None	1.40*	1.23-1.58	1.16	0.88-1.53	1.66*	1.39-1.98
SES^1^	1.39*	1.22-1.57	1.14	0.86-1.51	1.64*	1.38-1.96
Hormonal contraceptives	1.39*	1.22-1.57	1.14	0.86-1.50	1.64*	1.38-1.96
HRT^5^	1.38*	1.22-1.57	1.14	0.86-1.50	1.63*	1.37-1.95
Parity	1.38*	1.22-1.56	1.14	0.86-1.50	1.62*	1.36-1.94

OR, odds ratio; CI, confidence interval;

*p < 0.05.

^1^Educational level, marital status and income;

^2^Self-reported somatic diagnoses and medication for somatic conditions;

^3 ^Somatic symptoms included in symptom criteria of the ICD-10 diagnosis F45.0 Somatization disorder (skin symptoms excluded);

^4^Including occupation/industry, omega-3 supplement and current smoking;

^5^Hormonal replacement therapy (Oestrogen tablets or Plaster).

Linear regression analyses, employing z-scored variables, showed that somatization and health anxiety were more strongly associated to eczema than anxiety and depression, (unstandardized regression coefficient (B) = 0.37 (p < 0.01) for the association between somatization and eczema, B = 0.22 (p < 0.01) for health anxiety/eczema, B = 0.20 (p < 0.01) for anxiety/eczema, and B = 0.15 (p < 0.01) for depression and eczema).

Adjustment for Fibromyalgia alone contributed to some extent and other somatic diagnoses (self-reported somatic diagnoses and medication for somatic conditions) explained a minor part of the association.

### Other covariates

Adjustments for gender, socio-economic factors, occupation and industry ("wet work" is included here), omega-3 supplement and current smoking did not reduce OR substantially (Table 3). Adjustments for hormonal contraceptives and replacement therapy and parity did not account for the association of interest (Table 3, lower part).

The association of interest (including candidate confounding/mediating factors) was also analyzed applying HADS as a continuous variable, and with linear regression models. All conclusions were confirmed applying this approach.

## Discussion

The present study confirmed previous findings of an association between anxiety and depression, and eczema [2-8,47-49]. But more important, the study adds to the current knowledge on the understanding of possible contributing factors in this association. Somatization and health anxiety accounted for about half of the association between anxiety/depression, and eczema, and the associations of interest were insignificant after this adjustment. Female gender and omega-3 fatty acid supplements have in psychodermatological literature been proposed as contributing factors relevant for the association of between anxiety, depression, and eczema, but we were unable to provide empirical support for these hypotheses.

The present study has several strengths, one of them being the epidemiological population-based design avoiding selection biases commonly found in clinical contexts.

Among the most important limitations to our study is the self-report of eczema, which precludes sub-classification beyond report of body area involved. Theoretically, several forms of eczema could be included in the eczema group due to the general form of the eczema questions used in our study. It is not uncommon, however, to employ a wider definition of eczema in large epidemiological studies. In a study by Hanifin and Reed, [50] the term "empirical eczema" was defined by several skin symptoms that resemble the skin symptoms listed in the HUSK questionnaire used in our study. Further, the validity of several self-reported skin complaints against clinical signs was explored in a Norwegian, population-based, validation study [51], finding that self-reported skin complaints can be a valid tool for quantifying and exploring skin diseases at population level. The use of self-reported eczema represents a risk of misclassification (both false positive and false negative cases compared to the result of a clinical examination), resulting in an underestimation of the true strength of the association. Meding and colleagues reported good agreement between patients' self-report of hand eczema and dermatologists' judgements [37,38]. Validation of the question "have you had hand eczema on any occasion during the last 12 months" indicated false negative answers among 10-12.5% of the participants [38]. This population survey is comparable to our present study, indicating similar extent of misclassification.

Another objection to our study, could be that hand eczema is a singular clinical entity, and that the reliability and validity of self-reported hand eczema cannot be generalized to eczema in other areas of the body. However, we have no reason to assume the validity to be poorer for other forms of self-reported eczema. In our analyses we compared face, hand and body eczema and their association to mental health, finding that the association was not restricted to special sub-types of eczema. Future studies of the validity of self-reported face and body eczema might be warranted, but while awaiting these, we have to assume that the validity of self-reported hand eczema can be generalized to other body areas.

The use of HADS for assessment of anxiety and depression also implies self-report rather than clinical examination. Misclassification cannot be excluded, but is likely to have been random; again resulting in an underestimation of the true association.

Another important limitation to our study is the use of a cross-sectional design, precluding inferences regarding causality for the association between anxiety and depression, and eczema. Employing a cross-sectional design, it is not possible to determine whether psychological symptoms can result from eczema, or if such symptoms are part of the causative chain for skin disease.

How can we interpret the finding that somatization and health anxiety explain most of the association between anxiety and depression, and eczema? One possibility is that health anxiety might represent selective attention to common somatic symptoms [52-54], which again might increase self-report of eczema. An alternative explanation could be that anxiety, depression, health anxiety and somatization are all a consequence of the burden of suffering from chronic eczematous conditions. Another explanation involves the possibility of a common factor in anxiety, depression, health anxiety and somatization which are correlated to eczema. In this perspective, our finding that somatization and health anxiety account for much of the association between anxiety/depression, and eczema might be regarded an artefact resulting from over-adjustments. However, the finding that somatization is more strongly associated to eczema than anxiety/depression makes us speculate that the association between anxiety/depression, and eczema is actually driven by somatization. Further, the large confounding effect of the somatization variable might indicate that it actually (at least partly) represents somatization, as primary adjustment for somatic diagnoses hardly attenuated the association. This does obviously not equivalence to clinical examinations. Adjustment for somatic diagnoses prior to symptoms of somatization, in the association of interest, was our attempt to exclude organic aetiology in somatization. Using an index based on questions derived from the somatization diagnostic criteria doesn't have the same validity as employing a validated instrument, but to the best of our knowledge, there is yet no well-validated and widely used instrument for somatization. Acknowledging the limitations of our measure; adjustment for somatization (on top of somatic diagnoses) explained about forty percent of the association of interest.

According to Koblenzer [55] the common origin of the skin and the central nervous system (CNS) from the embryonic neuro-ectoderm could explain the ongoing two-way physiologic communication between emotional input, CNS functioning, and cutaneous expression. Continuing Koblenzer's line of thought, it might be speculated that some forms of eczema and somatization may be mediated through a common immunological factor. A possible common immunological factor in eczema and somatization could be cytokines, which are known to be involved in the immunological mechanisms underlying eczema [56] and suggested by Dantzer [57] to underlie somatization. Dantzer suggests that stress (psychological included) can induce sensitization of the cytokine system in the brain, which further may lead to symptoms of mood disorders (anxiety and depression), and expression of somatic symptoms that resemble those seen in somatization [57]. According to ICD-10 [20], some skin symptoms (skin spots and discoloration of the skin) are already included in the symptom list for F45, somatization disorder. So, another possibility could be that somatization may lead to psychological stress reactions in the body, and psychological stress has been found to worsen or trigger outbursts of eczema [1].

Omega-3 fatty acid intake is also hypothesized to account for the association of interest [7,12,13], but our results provide no support for this hypothesis. It might be objected that omega-3 fatty acid intake was measured by self-report of dietary supplements (cod liver oil) only, and in fairly moderate doses, further omega-3 fatty acid content in regular diet was not accounted for. There are obvious limitations to our omega-3 fatty acid measure, possibly reducing expected effect sizes. However, if omega-3 fatty acid intake was a major factor in explaining the association, we would expect at least some attenuation of the association of interest. Further, we cannot exclude the possibility that a higher daily intake of omega-3 fatty acids than reported in this study is needed to demonstrate a mediating effect.

A gender-specific association between eczema and depression has been suggested by Timonen et al. [7,13,26]. Stratified analyses by gender, and testing of the interaction-term eczema by gender did not support this hypothesis, even if one significant gender-interaction was found across 12 analyses, but in this analysis the association between hand eczema and depression was stronger in men than in women, the opposite of Timonens hypothesis.

In the light of an epidemiological population-based study we have been able to examine several of the suggested contributing factors in the association between anxiety/depression, and eczema. The lack of support for different candidate explanations for the association of interest might be somewhat surprising. We note that somatization and health anxiety accounted for most of the association. The literature on somatization in eczema is scarce, and should be examined more thoroughly in future studies. Beyond the candidate explanatory factors discussed here, we are left with speculations as to biological mechanisms involved. Future research into the aetiology underlying the association between anxiety and depression, and eczema might benefit from including the concept of somatization and health anxiety.

## Conclusions

The current study confirmed previous findings of an association between anxiety and depression, and eczema, but somatization and health anxiety accounted for more than half of the association of interest, and the association was insignificant after this adjustment.

We found no support for previously suggested contributing factors in the association between anxiety/depression, and eczema such as omega-3 fatty acid supplement and female gender.

## Competing interests

The authors declare that they have no competing interests.

## Authors' contributions

AM planned and supervised the study. MK planned, drafted and consecutively revised the manuscript. MK and AM performed the statistical analyses. KGG and AM critically revised the manuscript. All authors read and approved the final manuscript.

## Pre-publication history

The pre-publication history for this paper can be accessed here:

http://www.biomedcentral.com/1471-5945/10/3/prepub
